# The role of 15 lipoxygenase 1 in asthma comes into focus

**DOI:** 10.1172/JCI155884

**Published:** 2022-01-04

**Authors:** Joshua A. Boyce

**Affiliations:** Division of Allergy and Clinical Immunology, Department of Medicine, Brigham and Women’s Hospital, Boston, Massachusetts, USA.

## Abstract

IL-4– and IL-13–driven epithelial cell expression of 15 lipoxygenase 1 (15LO1) is a consistent feature of eosinophil-dominated asthma known as type 2–high (T2-high) asthma. The abundant soluble products of arachidonic acid (AA) metabolized by 15LO1 reflect a high level of enzymatic activity in asthma and chronic rhinosinusitis. However, the precise role of 15LO1 and its products in disease pathogenesis remains enigmatic. In this issue of the *JCI*, Nagasaki and colleagues demonstrate a role for 15LO1 in controlling redox balance and epithelial homeostasis in T2-high asthma by metabolizing AA that is esterified to membrane phospholipids. The findings may pave the way toward the development of 15LO1 inhibitors as asthma treatments.

## Altered epithelial cell differentiation in chronic respiratory tract inflammation

Asthma and chronic rhinosinusitis with nasal polyposis (CRSwNP) are prevalent immune-mediated diseases of the respiratory tract that frequently coexist and cause substantial morbidity (1–3). CRSwNP and asthma are most often associated with eosinophilic respiratory tissue infiltration, mast cell hyperplasia, goblet cell metaplasia, and disrupted epithelial integrity (4), processes that are linked to the actions of type 2 (T2) cytokines (type 2 inflammation; ref. 5). Specifically, cytokines IL-4 and IL-13 alter epithelial cell differentiation trajectories and function while strongly inducing the expression of a cassette of characteristic transcripts (e.g., *MUC5AC*, *CLCA1*, *CCL26*, *POSTN*, and *INOS*) in local stromal cells by signaling though the α subunit of the IL-4 receptor (IL-4Rα) (6, 7). The strength of expression of these IL-4Rα–driven transcripts in bronchial and nasal mucosal epithelial cells correlates with indices of disease severity (8, 9). Moreover, the efficacy of a humanized monoclonal antibody against IL-4Rα (dupilumab) in the treatment of severe T2-high asthma and CRSwNP validates the biological importance of IL-4Rα–inducible products in disease pathophysiology (10, 11), although the relative contributions of each remain less clear.

## Pro- and antiinflammatory functions of 15LO1 products

*ALOX15*, encoding 15 lipoxygenase 1 (15LO1), is one of the strongly and consistently expressed IL-4Rα–inducible transcripts by mucosal epithelial cells in T2-high asthma and CRSwNP (7, 9, 12). Its expression is especially strong in aspirin-exacerbated respiratory disease (AERD), a disease phenotype that is overrepresented in the most severely affected patients with asthma and CRSwNP (9). *ALOX15* is also expressed inducibly by macrophages and constitutively by eosinophils (13). 15LO1 catalyzes the oxidation of arachidonic acid (AA) and other polyunsaturated fatty acids (PUFAs), forming peroxy derivatives that are precursors of diverse mediators. In contrast to other lipoxygenase enzymes (e.g., 5LO, 12LO), 15LO1 introduces molecular oxygen species into both free PUFAs and into PUFAs that are esterified in phosphatidylethanolamine (PE) in cell membranes (14). 15LO1 converts free AA to an unstable hydroperoxide, 15-OOH eicosatetraenoic acid (HpETE), which is rapidly reduced to 15(*S*)-HETE. These intermediates are converted in turn to diverse metabolites, including 5-oxo-15(*S*)-hydroxyeicosatetraenoic acid [5-oxo-15(*S*)-HETE], a chemoattractant for human eosinophils (15), eoxin C_4_, a conjugate of 15-HETE to reduced glutathione (GSH) that is generated by eosinophils (13), and other less-well-characterized products. 15LO1 also converts the granulocyte-derived 5LO product leukotriene A_4_ to lipoxin A_4_, a mediator that resolves inflammation (16). 15LO1 products abound in biological fluids (e.g., nasal lavage, bronchoalveolar lavage [BAL] from patients with asthma and CRSwNP; ref. 12), reflecting 15LO1 activity in vivo, and correlating with disease severity. Understanding the balance of pro- and antiinflammatory functions of 15LO1 products is essential to therapeutic targeting of this enzyme.

**Figure 1:**
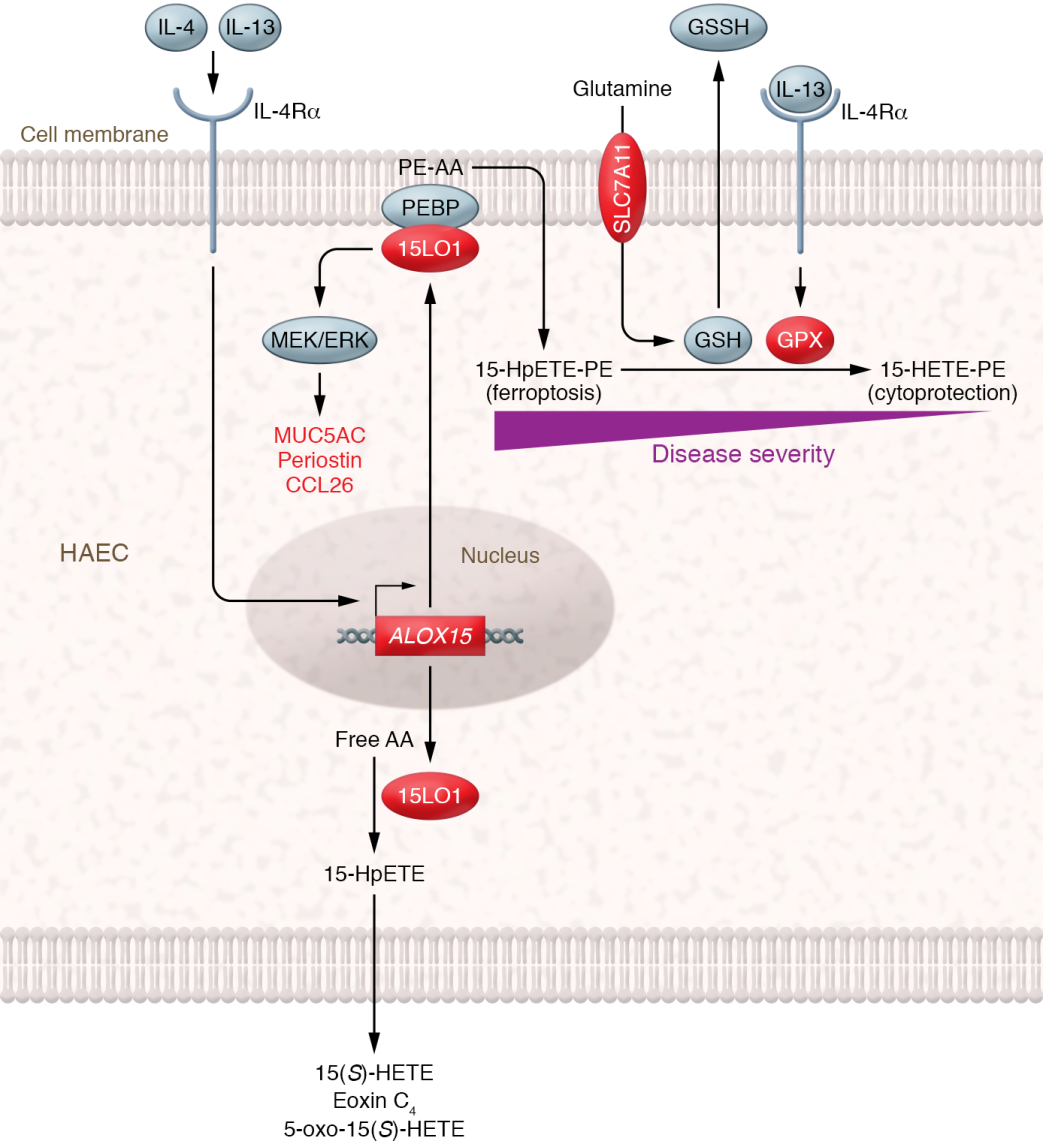
Model for dual role of 15LO1 in asthma related to type 2 inflammation. IL-4 and IL-13 induce IL-4Rα–dependent signaling to drive *ALOX15* (15LO1) expression in human airway epithelial cells (HAECs). 15LO1 can add molecular oxygen to free AA, forming HpETE that is rapidly reduced to 15(*S*)-HETE, which in turn can be converted into 5-oxo-15(*S*)-HETE, eoxin C_4_, and other detectable mediators found in biological fluids. 15LO1 also associates with PEBP in the cell membrane, activating ERK (leading to potentiated expression of additional type 2 inflammatory proteins) and switching the 15LO1 substrate preference to PE-esterified AA. The resultant HpETE-PE triggers ferroptosis unless it is reduced by GPX. This reduction requires GSH, which is maintained by the glutamic acid transporter protein SLC7A11. IL-13 increases both GPX and SLC7A11 expression, suggesting that a coordinated system maintains cellular redox balance. Perturbations in this system, such as diminished GSH availability, may favor ferroptosis over cytoprotection, leading to epithelial damage. Red color indicates proteins that are upregulated in epithelial cells by IL-4 and IL-13.

While soluble products of 15LO1 activity reflect peroxidation of free PUFAs, oxidative products of esterified PUFAs remain cell associated and are therefore more challenging to study. Both 15LO1 and its homologue, 15LO2 (which is constitutively expressed in several organs), interact with cell membrane–associated PE-binding protein (PEBP), a scaffolding protein that regulates mitogen-activated protein kinase (MAPK) cascades (17). The interaction between PEBP and 15LO1 permits IL-13 to induce the activation of extracellular signal–regulated kinase (ERK) (18). Additionally, the association with PEBP switches the substrate preference of 15LO1 from free to esterified AA, resulting in the formation of 15-HpETE esterified onto PE (HpETE-PE). Importantly, 15LO1-derived HpETE-PE is a potent trigger of ferroptosis (19), a form of iron-dependent programmed cell death involving lipid peroxidation (20). Glutathione peroxidase 4 (GPX4) reduces PE-associated lipid peroxides (21) and prevents ferroptosis by converting 15-HpETE-PE to 15-HETE-PE, consuming GSH in the process. Diminished expression of GPX4 or insufficient GSH levels alter redox balance, favoring ferroptosis in the context of 15LO1-PEBP interactions (19), a mechanism that may potentially disrupt epithelial function and barrier integrity (Figure 1).

## Controlling redox balance in the airway

In this issue of the *JCI*, Nagasaki and colleagues provide direct evidence that 15LO1-derived 15-HpETE-PE plays a role in controlling redox balance in the airway of patients with asthma, with potential pathophysiologic consequences (22). Using measures of redox balance in BAL fluids and freshly harvested bronchial epithelial cells from subjects with asthma who were enrolled in two cohort studies, the investigators found higher glutathione disulfide (GSSH) (reflecting the consumption of GSH) and lower GSH/GSSH ratios in BAL fluids from subjects with severe asthma than those with mild/moderate disease and healthy controls. Intracellular GSH levels and GSH/GSSH ratios were lowest in epithelial cells from severe asthmatic subjects, consistent with increased utilization of GSH to maintain homeostasis. Both BAL fluid and intracellular GSH/GSSH levels correlated inversely with the levels of exhaled nitric oxide, a surrogate marker of T2-driven *INOS* expression, and correlated positively with measures of lung function. These observations are consistent with altered redox balance in severe asthma that correlates with both physiologic impairment and with surrogate measures of T2 inflammation.

To understand the potential role of IL-4Rα–driven 15LO1 activity in altering epithelial redox balance, the authors treated cultured bronchial epithelial cells with IL-13 ex vivo. As expected, IL-13 strongly upregulated 15LO1 protein expression. Unexpectedly, IL-13 also upregulated the expression of both GPX4 and SLC7A11, a glutamine transporter necessary to maintain intracellular GSH, suggesting a coordinated cellular response to preserve redox homeostasis. Stimulation of the cultured cells with IL-13 decreased both intracellular and extracellular levels of GSH, increased extracellular GSSH levels, and decreased GSH/GSSH ratios in both compartments. Based on studies using small interfering RNA knockdown and pharmacologic inhibition of 15LO1 in IL-13–stimulated epithelial cells, the changes involved 15LO1. Intracellular GSH levels and GSH/GSSH ratios in freshly obtained bronchial epithelial cells correlated inversely with 15LO1 expression levels but positively with the SLC7A11/15LO1 expression ratio (22). Treatment of the IL-13–stimulated epithelial cells with erastin, an inhibitor of SLC7A11 that depletes intracellular levels of GSH, induced cell death while modestly increasing expression and secretion of CCL26, periostin, and MUC5AC, each of which had previously been linked to 15LO1 activity based on ex vivo studies (18, 22, 23, 24). Thus, perturbations in redox homeostasis may substantially influence the physiological consequences of induced 15LO1 expression in asthma, CRSwNP, and other diseases associated with T2 inflammation.

The study by Nagasaki et al. (22) directly implicates altered redox balance in severe asthma, and links this altered balance to the induced expression and function of 15LO1. Although the authors do not directly demonstrate 15LO1-mediated ferroptosis in vivo, it is tempting to speculate that the altered barrier integrity observed in both asthma and CRSwNP may at least partly reflect this process. While no currently available drugs block 15LO1 activity in humans, a loss-of-function mutation of *ALOX15* confers strong protection against the development of CRSwNP in cohorts from Sweden and the United Kingdom (25), strongly suggesting a key role for 15LO1 and its products in IL-4Rα–driven epithelial functional changes, and potential for therapeutic targeting of 15LO1 with inhibitors. It is tempting to speculate that the success of dupilumab as a treatment for asthma and CRSwNP could at least partly reflect a restoration of epithelial function by preventing ferroptosis (and other epithelial responses) that 15LO1 may control or influence.

## Version 1 01/04/2022

Electronic publication
